# PyMVPD: A Toolbox for Multivariate Pattern Dependence

**DOI:** 10.3389/fninf.2022.835772

**Published:** 2022-06-23

**Authors:** Mengting Fang, Craig Poskanzer, Stefano Anzellotti

**Affiliations:** Department of Psychology and Neuroscience, Boston College, Boston, MA, United States

**Keywords:** multivariate pattern dependence, connectivity, fMRI, deep networks, toolbox

## Abstract

Cognitive tasks engage multiple brain regions. Studying how these regions interact is key to understand the neural bases of cognition. Standard approaches to model the interactions between brain regions rely on univariate statistical dependence. However, newly developed methods can capture multivariate dependence. Multivariate pattern dependence (MVPD) is a powerful and flexible approach that trains and tests multivariate models of the interactions between brain regions using independent data. In this article, we introduce PyMVPD: an open source toolbox for multivariate pattern dependence. The toolbox includes linear regression models and artificial neural network models of the interactions between regions. It is designed to be easily customizable. We demonstrate example applications of PyMVPD using well-studied seed regions such as the fusiform face area (FFA) and the parahippocampal place area (PPA). Next, we compare the performance of different model architectures. Overall, artificial neural networks outperform linear regression. Importantly, the best performing architecture is region-dependent: MVPD subdivides cortex in distinct, contiguous regions whose interaction with FFA and PPA is best captured by different models.

## 1. Introduction

Cognitive processes recruit multiple brain regions. Understanding which of these regions interact, and what computations are performed by their interactions, remains a fundamental question in cognitive neuroscience. In an effort to answer this question, a large literature has used measures of the statistical dependence between functional responses in different brain regions. The most widespread approach adopted in this literature—“functional connectivity”—computes the correlation between the timecourses of responses in different brain regions, and has been applied to both resting state fMRI and task-based fMRI (Horwitz et al., 1992; Friston, 1994; Greicius et al., 2003; Schaefer et al., 2018). Other approaches, such as Granger Causality (Granger, 1969; Goebel et al., 2003) and Dynamic Causal Modeling (Friston et al., 2003), have been developed to investigate the directionality of interactions.

In a separate literature, researchers studying the content of neural representations have developed techniques that leverage the multivariate structure of activity patterns (multivariate pattern analysis—MVPA) to decode information from fMRI data (Norman et al., 2006), and to study the similarity between the responses to different stimuli (Kriegeskorte et al., 2008). The success of MVPA has inspired the development of multivariate approaches to study the statistical dependence between brain regions (Anzellotti and Coutanche, 2018; Basti et al., 2020).

One approach, “Informational Connectivity,” computes the trial-by-trial decoding accuracy for a given categorization in multiple regions, and correlates the decoding accuracy achieved with data from one region with the accuracy achieved with the other region across trials (Coutanche and Thompson-Schill, 2013). Another approach uses multivariate distance correlation to capture the statistical dependence between regions (Geerligs et al., 2016)—thanks to this strategy, it can also be applied to resting state studies, in which different conditions that can be categorized are not available.

Among multivariate approaches to study the interactions between brain regions, multivariate pattern dependence (MVPD, Anzellotti et al., 2017a; Li et al., 2019) is unique in that it trains and tests models of the interactions between brain regions using independent subsets of data, evaluating out-of-sample generalization. Like multivariate distance correlation, MVPD can be applied to both task data and resting state data. Additionally, MVPD can flexibly use a variety of models of dependence, with the potential to incorporate regularization, and to capture linear, as well as non-linear, interactions between brain regions. Of course, the use of holdout data for model evaluation has been previously adopted for applications outside the field of connectivity—indeed, it is also used in MVPA (Haxby et al., 2001; Haynes and Rees, 2006), and it has an even longer history in machine learning (see for instance Lachenbruch and Mickey, 1968). Similarly, the use of multivariate methods is also present in MVPA, and has a longer history in Science (Pearson, 1903).

Given the complex nature of the MVPD, a dedicated toolbox can provide researchers with a more accessible entry point to adopt this method. Several toolboxes have been developed for MVPA (Hanke et al., 2009; Hebart et al., 2015; Oosterhof et al., 2016; Treder, 2020), these toolboxes played an important role for the diffusion of MVPA analyses (as evidenced by the many times they have been cited). Here, we introduce a freely available open-source toolbox for MVPD, developed in Python: PyMVPD. The toolbox offers a set of functions for performing MVPD analyses, organized around a simple workflow. It also includes example Python scripts for several MVPD models, including linear regression models that were used in previous MVPD publications (Anzellotti et al., 2017a; Li et al., 2019), and new models based on artificial neural networks. The models are accompanied by algorithms that can be used to evaluate their performance. PyMVPD scripts have been designed so that they can be easily customized, enabling users to expand the toolbox to address their needs.

The full PyMVPD toolbox (including the artificial neural network models) requires a working installation of PyTorch. The use of CUDA and general purpose graphics processing units (GPGPUs) is recommended. For users who might not need artificial neural networks, we also make available a lite version of the toolbox, that does not require PyTorch. Both versions of PyMVPD can be installed with PyPI.

**Figure 1:**
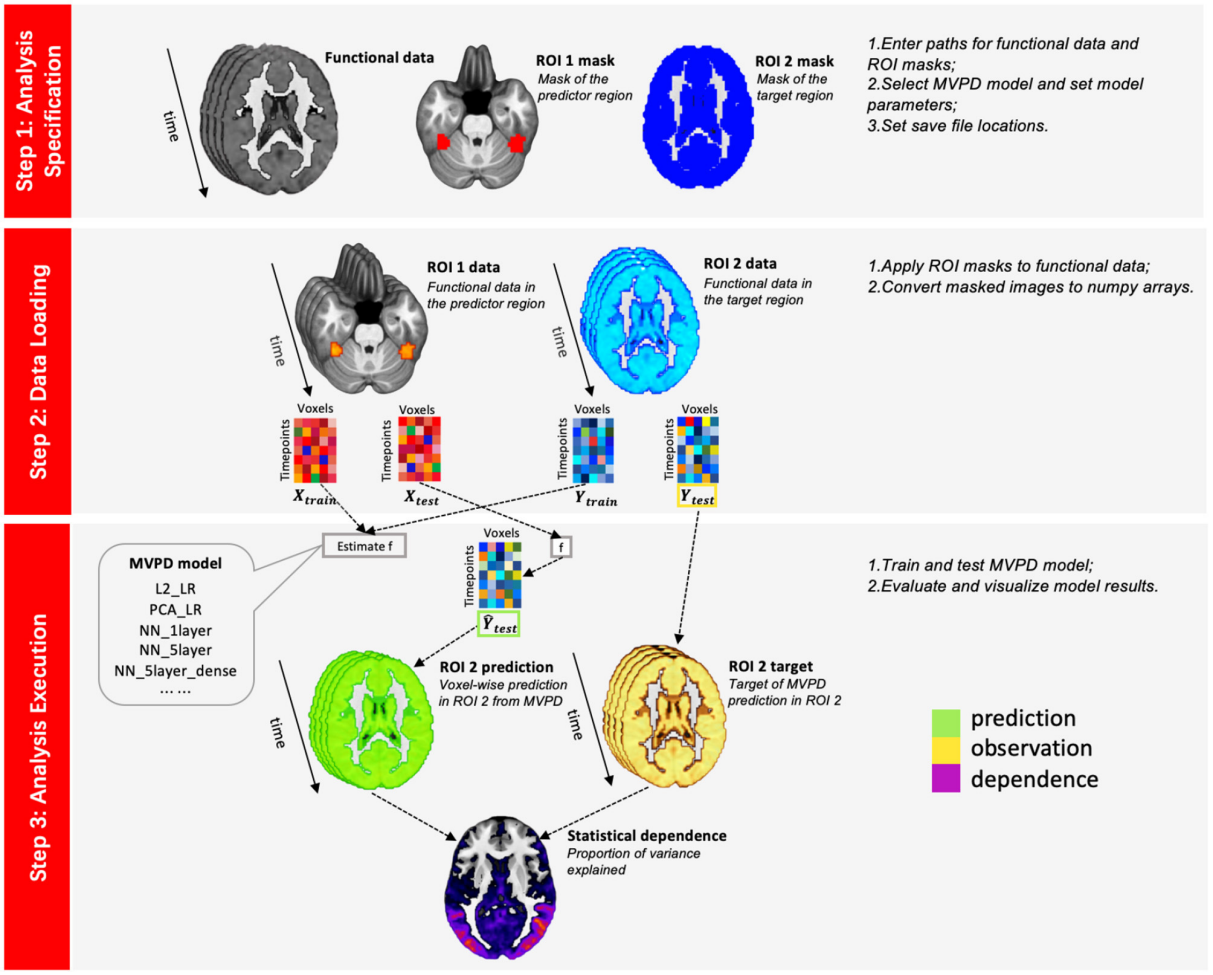
PyMVPD workflow. Analyzing data with the PyMVPD toolbox consists of three steps: (1) analysis specification; (2) data loading; and (3) analysis execution. In step 1, users are required to specify the functional data, masks for both the predictor and the target region, as well as the type of MVPD model to perform the following analyses. These details can be specified by editing the Python script *run_MVPD.py*. Next, users can proceed with step 2, which loads neuroimaging data and converts it to a suitable format. Finally, step 3 runs the MVPD model and generates the analysis results, which are saved in a user-specified directory.

In the remainder of the article, a brief technical introduction to MVPD is followed by a description of PyMVPD implementation and the analysis workflow (Figure 1). Next, the algorithms are validated by analyzing a publicly available dataset—the StudyForrest dataset (Sengupta et al., 2016). Finally, the performance of different types of models is assessed, comparing the predictive accuracy of linear regression and artificial neural networks.

## 2. Methods

### 2.1. MVPD

Multivariate pattern dependence (MVPD) is a novel technique that analyzes the statistical dependence between brain regions in terms of the multivariate relationship between their patterns of response. Compared with traditional methods used for connectivity analysis, MVPD has two main advantages (Anzellotti et al., 2017a). First, MVPD preserves the fine-grained information that can be lost by spatially averaging in mean-based univariate connectivity. By doing so, MVPD improves sensitivity as compared to univariate methods such as standard functional connectivity (Anzellotti et al., 2017a). This choice is motivated by the success of multivariate analysis methods developed outside the field of connectivity (MVPA, Haxby et al., 2001; Haynes and Rees, 2006; Norman et al., 2006). Second, MVPD is trained and tested with independent subsets of data. As a consequence, it is resilient to overfitting: in MVPD it is not sufficient for a model of the interactions between two regions to provide a good fit for a set of data, the model also has to generate accurate predictions for a separate set of data that was not used to tune the model's parameters (the “testing” data). This is a key feature of MVPD: it guarantees a more stringent test of the interactions between brain regions. Note that however this procedure does not remove the need for denoising methods: some sources of noise can produce shared effects across multiple regions. A previous study investigated the effectiveness of different denoising techniques for MVPD (Li et al., 2019); among the techniques tested, CompCor (Behzadi et al., 2007) was the most effective, therefore we used CompCor for denoising in this study.

Due to the use of separate sets of data for training and testing, MVPD benefits from fMRI datasets that include multiple experimental runs within each participant. This way, there is a sufficient amount of data to train the models, even after holding some out for testing. The amount of training data within a participant affects the model's ability to generate accurate predictions for the testing data. For this reason, datasets that include a very short amount of data within each participant (e.g., a 5-min resting state scan) are not well-suited for this type of analysis—in this respect, MVPD is similar to multivariate pattern analysis (MVPA).

The number of participants needed for MVPD analysis might vary depending on the brain regions that are being investigated. In previous studies, numbers of participants similar to the ones used for MVPA have produced robust results (Anzellotti et al., 2017a; Li et al., 2019). Based on these considerations, in the present work we used the StudyForrest dataset (Hanke et al., 2016), a publicly available dataset that has been used in several MVPA studies. As compared to large datasets used in functional connectivity (such as the Human Connectome Project dataset, Smith et al., 2013), the number of participants in the StudyForrest dataset is relatively small (14 subjects for analysis), but StudyForrest includes over 2 h of data for each individual participant, making it ideal for MVPD.

The logic of MVPD is as follows. Suppose that we want to calculate the statistical dependence between two brain regions. MVPD will learn a function that, given the response pattern in one region (the “predictor” region), generates a prediction of the response pattern in the other (the “target” region). Let us consider an fMRI scan with *m* experimental runs. We denote the multivariate timecourses in the predictor region by *X*_1_, …, *X*_*m*_. Each matrix *X*_*i*_ is of size *n*_*X*_ × *T*_*i*_, where *n*_*X*_ is the number of voxels in the predictor region, and *T*_*i*_ is the number of timepoints in the experimental run *i*. Analogously, *Y*_1_, …, *Y*_*m*_ denote the multivariate timecourses in the target region, where each matrix *Y*_*i*_ is of size *n*_*Y*_ × *T*_*i*_, and *n*_*Y*_ is the number of voxels in the target region.

As a first step, the data is split into a training subset and a test subset. It is important that the training and test subsets are independent. Since fMRI timeseries are characterized by temporal autocorrelation, it is best to not use timepoints from one run for training and adjacent timepoints from the same run for testing. A common approach is to use leave-one-run-out cross-validation: this is the approach implemented by default in the PyMVPD toolbox. For each choice of an experimental run *i*, data in the remaining runs is concatenated as the training set


$$\begin{array}{l} D_{i}=\left\{\left(X_{1},Y_{1}\right),...,\left(X_{i-1},Y_{i-1}\right),\left(X_{i+1},Y_{i+1}\right),...,\left(X_{m},Y_{m}\right)\right\}, \end{array}$$

while data *D*_*i*_ = {(*X*_*i*_, *Y*_*i*_)} in the left-out run *i* is used as the test set.

For convenience, we will denote with *X*_*train*_ and *Y*_*train*_ the concatenated training data in the predictor region and in the target region, respectively. The training data is used to learn a function *f* such that


$$\begin{array}{l} Y_{train}=f\left(X_{train}\right)+E_{train}, \end{array}$$

where *E*_*train*_ is the error term. In the current implementation, the response pattern in the target region at a given time is predicted from the response pattern in the predictor region at the same time. However, models that integrate the responses in the predictor region across multiple timepoints are a straightforward extension. Once the function *f* has been estimated, we use it to generate predictions of the responses in the target region Ŷ_*test*_ given the responses in the predictor region during the test run:


$$\begin{array}{l} {Ŷ}_{test}=f\left(X_{test}\right). \end{array}$$

Finally, the accuracy of the prediction is computed. In the PyMVPD toolbox, we provide a measure of predictive accuracy by calculating the voxelwise proportion of variance explained. For each voxel *j* in the target region, variance explained is calculated as:


$$\begin{array}{l} \text{varExpl}\left(j\right)=1-\frac{var\left[Y_{test}\left(j\right)-{Ŷ}_{test}\left(j\right)\right]}{var\left[Y_{test}\left(j\right)\right]}, \end{array}$$

where Ŷ are the predicted voxelwise timecourses. The values varExpl(*j*) are then averaged across voxels in the target region and across cross-validation runs to obtain a single measure varExpl. In addition, the PyMVPD toolbox is designed to allow for customized measures of accuracy (more details will be provided in the following sections).

### 2.2. The PyMVPD Toolbox

PyMVPD is a Python-based toolbox that implements the MVPD analysis pipeline. This software package is freely available at https://github.com/sccnlab/PyMVPD.git. Artificial neural network models are built using PyTorch—for users who are only interested in linear regression models, or who would like to avoid the complexities of a PyTorch installation, we have also provided a lite version (PyMVPD_LITE) at https://github.com/sccnlab/PyMVPD_LITE.git, for which PyTorch is not required. PyMVPD is based on a simple workflow that consists of three steps: analysis specification, data loading, and analysis execution, with the latter step including the following sub-steps: dimensionality reduction (if requested), model estimation, and model evaluation. Models are trained and tested using k-fold cross validation (where k is a parameter specified by the user).

#### 2.2.1. Preliminaries

Prior to MVPD analysis, the fMRI data at hand should have already undergone standard preprocessing steps, such as registration, normalization and denoising. Denoising is an essential component of preprocessing: measures of statistical dependence are susceptible to noise (Ciric et al., 2017). The preprocessed fMRI data should be in NIfTI file format. Next, the user should create brain masks of the predictor region (“ROI 1”) and the target region (“ROI 2”), also in NIfTI file format.

#### 2.2.2. Step 1—Analysis Specification

During analysis specification, the user enters all necessary information to perform the analysis into the script “run_MVPD.py”. Information is organized into two variables: “inputinfo”, and “params”. The variable “inputinfo” contains the paths to the input data as well as the locations to which the results will be saved (the complete list of required values can be found here https://github.com/sccnlab/PyMVPD#required-input-information). The variable “params” contains all details about the analysis, including the type of cross-validation (e.g., leave-k-run-out), the type of dimensionality reduction chosen (if any), the number of dimensions selected, the type of model of statistical dependence, and other hyperparameters of the model (for example, the amount of regularization for regularized regression models, or the neural network architecture for neural network models). We include an overview of the key options available in the Section 2.2.4. A complete description of all parameters would not fit within the limits of this article, therefore it is reported at this page: https://github.com/sccnlab/PyMVPD#list-of-model-parameters (along with the default values for each parameter). Since all parameters for the analysis are specified by the user in step 1, before the analysis is launched, and all results and logs are automatically saved to a user-specified folder, PyMVPD jobs can be launched on computer clusters as batch jobs, without the need to use interactive jobs.

#### 2.2.3. Step 2—Data Loading

The second step of PyMVPD is the loading and processing of input data. Before running the chosen MVPD model, values from the functional data are extracted using masks specified in step 1, and transformed into numpy arrays in preparation for the following analyses. To accomplish this step, the user can execute the line of code *data_loading.load_data(inputinfo)*.

#### 2.2.4. Step 3—Analysis Execution

Once the analysis details have been specified and the data is loaded, the third step executes the analysis, estimating the statistical dependence between brain regions and reporting the accuracy of predictions in independent data. To perform step 3, users can call the function *model_exec.MVPD_exec(inputinfo, params)*, which will estimate the MVPD model, compute the model's performance, and save the results to the folder specified in step 1.

It is important to note that during the implementation of PyMVPD, users only need to interface with the analysis specification script in the first analysis step (e.g., run_MVPD.py). Then, the following two analysis steps will run automatically and the users are not required to interface with any of them. This default setting makes it easier to run the toolbox on computer clusters.

Logging information is saved as a text file named by “TIMESTAMP_log.txt” under the directory where users specify to save results in the first analysis step. The log file contains information about input data that have been used for the analysis, MVPD model parameters, and the version of the toolbox.

To ensure the toolbox is installed properly and to verify it works, we have included tests for users to run before performing formal analyses. Users can find the test script “run_MVPD_test.py” under the exp/ folder. The test script attempts to replicate on the user's machine the analyses in our manuscript that used FFA as seed using data from subject sub-01, and calculates for each of the five example models the correlation between the variance explained values we obtained and the values obtained with the user's installation across the whole brain. If the correlation values are below 0.95 for any of the model types, the test script returns a warning notifying the user that the results they obtained do not match the benchmarks, specifying which of the models produced results that differed from our reference results. The results of the tests are saved in the folder exp/testresults/, so that the tests can be executed as a batch script on a computer cluster.

Since executing all analyses can take a substantial amount of time, in addition to the “run_MVPD_test.py” script we have included scripts to test individual models. This way, users can test just the type of model they are interested in using. These tests for individual models are also included in the exp/ folder, with the names “run_MVPD_PCA_LR.py”, “run_MVPD_L2_LR.py”, “run_MVPD_NN_1layer.py”, “run_MVPD_NN_5layer.py”, and “run_MVPD_NN_5layer_dense.py”. In future extensions of the toolbox, we plan to introduce more finer-grained tests for individual functions.

Below, we provide an overview of the available options for the different stages of analysis execution (dimensionality reduction, model estimation, model evaluation). Users can select what options to use for their analysis by editing the file “run_MVPD.py” (as noted in the Section 2.2.4).

##### 2.2.4.1. Dimensionality Reduction

The PyMVPD toolbox offers the option to perform dimensionality reduction on the input data before estimating models of statistical dependence. Dimensionality reduction can be desirable because reducing the dimensionality of the input data leads to a corresponding reduction in the number of parameters of the models, mitigating the risk of overfitting. Two dimensionality reduction approaches are included in the toolbox: principal component analysis (PCA), implemented with sklearn.decomposition.PCA, and independent component analysis (ICA), implemented with sklearn.decomposition.FastICA. In the current implementation of the toolbox, the number of dimensions needs to be entered manually by the user (the default value is 3), but the toolbox is designed to accommodate custom dimensionality reduction functions, offering the possibility to include a nested cross-validation approach for the selection of the number of dimensions (this option may be implemented as a core part of the toolbox in future releases). In particular, for applications in which the choice of the number of dimensions has meaningful theoretical implications, we recommend implementing a custom PCA function that uses Minka's MLE algorithm to select the number of dimensions based on the data. For some model types, dimensionality reduction might not offer additional benefits. In particular, when using artificial neural network models, the neural networks can themselves perform dimensionality reduction as needed—the desired amount of dimensionality reduction can be regulated by choosing the appropriate size of the hidden layer (or layers). Hidden layers with a smaller number of hidden nodes correspond to greater data compression.

##### 2.2.4.2. Model Estimation

###### 2.2.4.2.1. Linear Regression Models

Linear regression attempts to model the relationship between a dependent variable and one or more explanatory variables by fitting a linear function to observed data. Specifically, we view the multivariate timecourses in the predictor region *X* as the explanatory variable and the multivariate timecourses in the target region *Y* as the dependent variable. The MVPD mapping *f* can be modeled with multiple linear regression


$$Y_{train}=B_{train}X_{train}+E_{train},$$

where *B*_*train*_ is the vector of parameters and *E*_*train*_ is the error vector.

A large number of parameters as compared to a relatively small dataset can lead regression models to overfit the data. That is, the model learns a function that corresponds too closely to the particular training set and therefore fails to fit unseen data, resulting in poor predictive accuracy during testing. To mitigate this issue, we provide the option to choose either Lasso or Ridge regularization, setting “params.reg_type” to either “Lasso” or “Ridge”. The strength of regularization can be either set manually using the parameter “params.reg_strength” (the default value is 0.001), or automatically thanks to the use of nested cross-validation (Golub et al., 1979). When choosing to set the regularization parameter manually, it is of fundamental importance to decide the value of the parameter a-priori. Performing the analysis with multiple choices of the regularization parameter and selecting the one that yields the best results is a form of circular analysis, and will lead to false positive inflation. To perform automatic selection, we offer the option to use Ridge regularization determining the regularization parameter with a nested cross-validation loop. This can be achieved setting “params.reg_type” to “RidgeCV”. By default, the optimal regularization value is chosen among 0.001, 0.01, and 0.1, users can specify a different set of regularization values to test by setting the parameter “params.reg_strength_list”. However, it is important to note that automatic selection of the regularization parameter may lead to longer computation times for the analyses.

###### 2.2.4.2.2. Neural Network Models

In addition to linear regression models, we introduce an extension of MVPD in which the statistical dependence between brain regions can be modeled using artificial neural networks. In this approach, the multivariate patterns of response in the predictor region are used as the input of a neural network trained to generate the patterns of response in the target region. In PyMVPD, all neural network models are trained using stochastic gradient descent (SGD) on a mean square error (MSE) loss by default. Batch normalization is applied to the inputs of each layer. Additionally, users should set the following hyperparameters for the chosen neural network: number of hidden units in each layer, number of layers, learning rate, weight decay, momentum, mini-batch size, and number of epochs for training. We provide standard fully-connected feedforward neural network architectures (“NN_standard”) and fully-connected feedforward neural network architectures with dense connections (“NN_dense”, Huang et al., 2017) for users to choose. Both architectures only consist of linear connections between layers without introducing non-linear activation functions. Users are welcome to build their own neural network models with customized functions.

Note that functional MRI data has temporal dependencies. That is, the amount of response in a voxel in a volume acquired at a given timepoint is not entirely independent of the amount of response in that voxel in the previous timepoint. This non-independence can potentially affect models of the interactions between brain regions, including standard functional connectivity as well as MVPD. In order to mitigate the effect of non-independence, the neural network models in PyMVPD adopt a strategy borrowed from deep Q-learning. In deep Q-learning there is a similar problem: the actions taken by a reinforcement learning agent, the resulting states of the environment, and the rewards are non-independent across adjacent timepoints. To mitigate this problem, actions and the resulting states are logged to a “replay memory”; the policy network is then trained on a batch sampled randomly from the replay memory, so that the actions, states and rewards in each batch are more independent (see Fan et al., 2020). PyMVPD neural network models use the same strategy: each training batch contains datapoints collected at a randomly sampled set of timepoints. As a consequence, the set of datapoints within a batch are more independent than if they had been sampled consecutively.

###### 2.2.4.2.3. Searchlight Analysis

Previous MVPD studies included searchlight-based analyses (Anzellotti et al., 2017a). The results of searchlight analyses can be contingent on the use of a sphere as the searchlight shape, and on the choice of a particular radius. To avoid this, we recommend using multi-output models instead: users interested in mapping the statistical dependence between one region and the rest of the brain can use a whole-brain mask as the target region (as we have done in the present work).

##### 2.2.4.3. Model Evaluation

To measure the predictive accuracy of the MVPD model after execution, we included code to calculate variance explained following two different approaches. In one approach, the variance explained values are left unthresholded, and thus can range between −∞ and 1. This can be helpful to identify cases in which there is a clear mismatch between the target and the prediction. However, since negative values of variance explained are difficult to interpret in terms of their neuroscientific implications, and since very negative outliers in individual participants can conceal voxels with positive variance explained in most participants, we additionally implemented a function to set negative variance explained to zero, indicating that the model failed to predict the responses in a given voxel for a given participant.

Notably, even when setting negative values of the variance explained to zero, the variance explained approach is more stringent than computing Pearson correlation between the model predictions and the observations. For example, in the presence of predictions that match the observations in terms of their patterns, but show a large difference in the means, Pearson correlation would be very high, while variance explained would be zero.

Statistical significance can be computed by performing a permutation test on the unthresholded variance explained values. Alternatively, phase resampling of the responses in the target of prediction could be used to construct the null distribution (see Liu and Molenaar, 2016). In addition, comparisons between the predictive accuracy of different predictor regions or different types of models can be done using non-parametric statistical tests on the differences between their proportions of variance explained (thresholded or unthresholded). This was the approach we adopted in the experimental application of PyMVPD in this article.

We assessed the statistical significance across participants with statistical non-parametric mapping (Nichols and Holmes, 2002) using the SnPM13 software, using FWE-correction at the voxel level to control for multiple comparisons (http://warwick.ac.uk/snpm). More specifically, to identify significant differences between two models, we first computed the average variance explained across cross-validation iterations for each voxel and for each model, and then we computed differences between these averages for the two models, obtaining one difference map for each participant. Finally, these difference maps were entered in SnPM13 following the steps described in this tutorial: https://warwick.ac.uk/fac/sci/statistics/staff/academic-research/nichols/software/snpm/man/exnew, and significance was computed selecting the option “MultiSub: One Sample *t*-test on diffs/contrasts”. When computing statistical significance, it is important to consider the spatial autocorrelation of fMRI data: the measurements from nearby voxels tend to be correlated. Some types of correction for multiple comparisons (e.g., cluster correction) can be susceptible to spatial autocorrelation, when using such methods, underestimating the spatial autocorrelation may lead to excessively liberal statistical thresholds. When there is not complete confidence that spatial autocorrelation can be correctly estimated, we recommend using thresholds corrected at the voxel level.

Importantly, if negative values of variance explained are set to zero, the use of standard statistical tests (such as *t*-tests) to establish significance can lead to exceedingly liberal thresholds. Recent work has investigated in depth this problem in the context of classification accuracy (Allefeld et al., 2016; Hirose, 2021), introducing new statistics that can be used to address this issue. Future work may lead to the development of approaches to implement FWE correction for these statistics, making it possible to apply them to whole-brain analyses controlling for multiple comparisons. In the meantime, we recommend either using raw variance explained values (without setting negative values to zero), or performing statistical tests on subtractions between the variance explained values obtained with different model types or different brain regions.

### 2.3. Application to Experimental fMRI Data

#### 2.3.1. Data Acquisition and Preprocessing

As a demonstration of the use of PyMVPD, we analyzed fMRI data of 15 participants (age range 21–39 years, mean 29.4 years, 6 females) watching a movie, from the publicly available *StudyForrest* dataset (http://studyforrest.org). Functional data were collected on a whole-body 3 Tesla Philips Achieva dStream MRI scanner equipped with a 32 channel head coil. The BOLD fMRI responses at the resolution of 3×3×3 mm were acquired using a T2^*^-weighted echo-planar imaging sequence. Complete details can be found in Hanke et al. (2016).

The dataset includes a movie stimulus session, collected while participants watched the 2-h audio-visual movie “Forrest Gump”. The movie was cut into eight segments, and each segment lasted approximately 15 min. All eight segments were presented to participants in chronological order in eight separate functional runs. Additionally, the dataset includes an independent functional localizer that can be used to identify category-selective regions (Sengupta et al., 2016). During the category localizer session, participants viewed 24 unique gray-scale images from each of six stimulus categories: human faces, human bodies without heads, small artifacts, houses, outdoor scenes, and phase scrambled images. Each participant was presented with four block-design runs and a one-back matching task.

All fMRI data was preprocessed using fMRIPrep (https://fmriprep.readthedocs.io/en/latest/index.html). Anatomical images were skull-stripped with ANTs (http://stnava.github.io/ANTs/), and segmented into gray matter, white matter, and cerebrospinal fluid using FSL FAST. Functional images were corrected for head movement with FSL MCFLIRT (https://fsl.fmrib.ox.ac.uk/fsl/fslwiki/MCFLIRT), and were subsequently coregistered to their anatomical scan with FSL FLIRT. Data of one participant was excluded because it could not pass the fMRIPrep processing pipeline. For the remaining 14 participants, we removed noise from the data with CompCor (Behzadi et al., 2007) using 5 principal components extracted from the union of cerebrospinal fluid and white matter. Regions of no interest for the cerebrospinal fluid and white matter were defined individually for each participant.

#### 2.3.2. ROI Definition

In each individual participant, seed regions of interest (ROIs) in the fusiform face areas (FFA) as well as the parahippocampal place areas (PPA) were defined using the first block-design run from the functional localizer. We performed whole-brain first level analyses on each participant's functional data by applying a standard general linear model with FSL FEAT (Woolrich et al., 2001). Next, we identified the peak voxels with the highest *t*-values for the contrast between the preferred category and other categories (i.e., FFA contrast: faces > bodies, artifacts, scenes, and scrambled images; PPA contrast: scenes > faces, bodies, artifacts, and scrambled images). We generated spheres of 9 mm radius centered in the peaks. Finally, the voxels within spheres from the left and right hemispheres were combined, and the 80 voxels with the highest *t*-values were selected (this is a common choice in neuroimaging studies, see Skerry and Saxe, 2014; Kliemann et al., 2018).

We additionally created a group-average gray matter mask using the gray matter probability maps generated during preprocessing, with a total of 53,539 voxels, that was used as the target of prediction.

#### 2.3.3. PyMVPD Analysis

Using the PyMVPD toolbox, we estimated the multivariate pattern dependence between each ROI (FFA/PPA) and the gray matter using five example MVPD models: **L2_LR**, **PCA_LR**, **NN_1layer**, **NN_5layer**, and **NN_5layer_dense**. **L2_LR** is a linear regression model with Ridge (L2) regularization. The regularization strength was set to be 0.001. **PCA_LR** is a linear regression model that applies dimensionality reduction on input data with PCA using three principal components. **NN_1layer** and **NN_5layer** are fully-connected feedforward neural networks derived from the “NN_standard” architecture with one hidden layer and five hidden layers, respectively. Under the “NN_dense” architecture, **NN_5layer_dense** is a fully-connected feedforward neural network with dense connections and five hidden layers. For all the neural network models, we set the number of hidden units in each hidden layer to be 100. Each network was trained with a batch size of 32, a learning rate of 0.001, and a momentum of 0.9 with no weight decay.

For both FFA and PPA, we took the 80 voxels in the seed ROI as the predictor region, and the 53,539 voxels in the gray matter as the target region. For each MVPD model, 7 of the 8 movie runs were used for training, and the remaining run was used for testing. This leave-one-run-out procedure was repeated 8 times by leaving aside each possible choice of the left-out run. We then calculated the variance explained for each voxel in the target region with all five MVPD models in the left-out data.

The proportion of variance explained for each seed region and model was computed for each voxel in gray matter, negative values of variance explained were set to 0. Next, we compared the overall predictive accuracy in each pair of MVPD models. For each participant, the proportion of variance explained by each model was averaged across all voxels in the gray matter, and across all cross-validation folds. The difference between the average variance explained by the two models was computed for each participant, and the significance was assessed with a one-tailed *t*-test across participants—*p*-values were Bonferroni corrected for all 20 comparisons (since one-tailed tests were used, comparisons in both directions were counted in the correction).

In addition to testing the models' overall predictive accuracy, we sought to compare their accuracy at the level of individual voxels. First, we performed a voxelwise comparison of neural network models vs. linear regression models. To do this, for each voxel, we calculated the average variance explained across neural network (NN) models, and we subtracted the average variance explained across linear regression (LR) models. We computed statistical significance across participants with statistical non-parametric mapping using the SnPM13 software, obtaining pseudo-t statistics for each voxel. Then, we identified voxels where neural network models significantly outperformed linear regression models, at a familywise error (FWE) corrected threshold of *p* <0.05 (voxelwise FWE-correction was used). Next, we performed finer-grained analyses, focusing on the better performing NN models. In particular, we tested all pairwise comparisons between individual NN models. As in the previous analysis, significance was computed using SnPM13, using a voxelwise FWE-corrected threshold of *p* <0.05. We used Bonferroni correction to control for the number of multiple comparisons.

Even in regions where different models are not significantly different, qualitative differences might reveal large-scale patterns that could help users in the selection of a particular model. Given this consideration, we aimed to provide a qualitative evaluation of the relative performance of different models across the brain. To this end, for each voxel in the gray matter, we first selected the model yielding the highest proportion of variance explained in that voxel (averaged across participants) and specified that model as the best model for that voxel. Then, we obtained a conservative measure of the extent to which the model outperformed the other models by calculating the lowest *t*-value among all comparisons between the best model and all other models. As these results are qualitative in nature, they are shown in the Supplementary Material (Supplementary Figure 6).

## 3. Results

**Figure 2:**
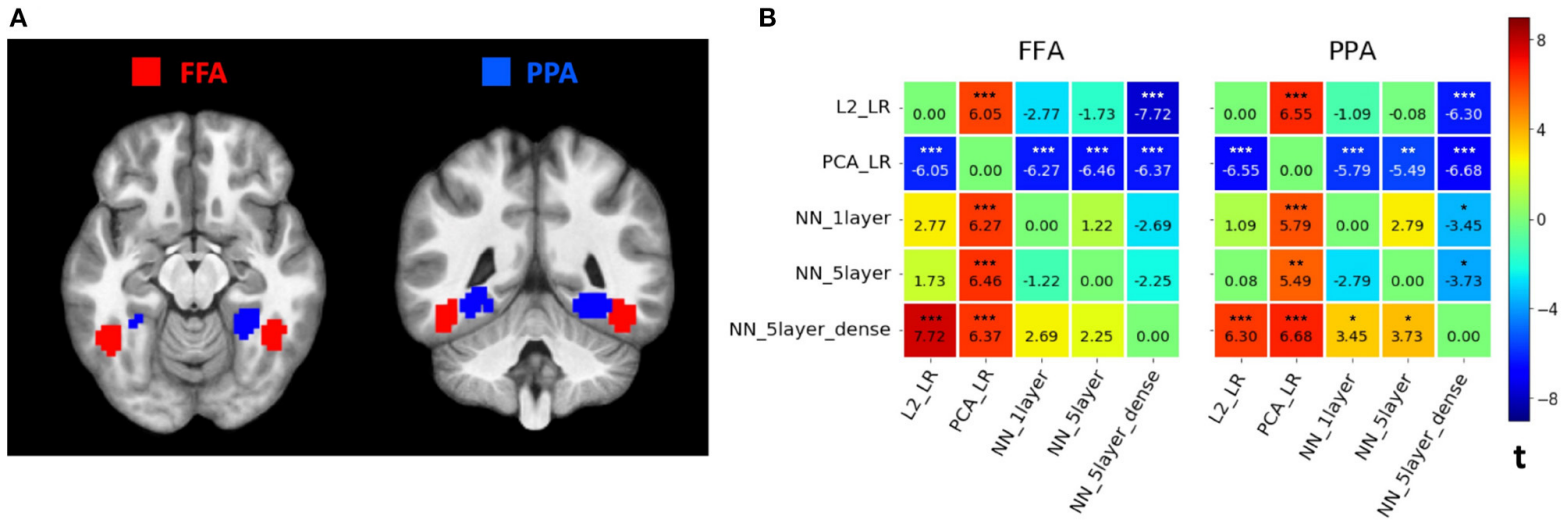
**(A)** Seed regions. Example seed regions for one representative participant. The fusiform face areas (FFA) are shown in red, and the parahippocampal place areas (PPA) are shown in blue. Individual ROIs per participant were defined based on first-level t-maps, identifying 9 mm spheres centered on the peaks for the preferred category, and selecting the top 80 voxels with highest *t*-values within the spheres. **(B)** Comparison between different MVPD models. For each seed predictor region (left: FFA; right: PPA), we plotted the difference matrix of *t*-values across the five example MVPD models. As a measure of overall predictive accuracy, the average proportion of variance explained varExpl was computed across the whole brain for each model per participant. For each pair of models, we subtracted the varExpl of one model from another one, obtaining the pairwise difference values. Finally, we conducted a one-tailed *t*-test on the difference values across all 14 participants. The corresponding *t*-values were entered into the difference matrix, indicating the extent to which one model outperformed another one in terms of the overall predictive accuracy using the seed region as the predictor. Stars above *t*-values indicate the statistical significance (***p <0.001;**p <0.01;*p <0.05; Bonferroni-corrected).

In line with previous studies using MVPD (Anzellotti et al., 2017a), our implementation of PyMVPD identified the expected patterns of statistical dependence between FFA and PPA and other brain regions (see Figure 2A for a visualization of the seed regions in one representative participant). Across multiple model types, when using FFA as seed, regions showing high variance explained included other face-selective regions, and when using PPA as seed, regions showing high variance explained included other scene selective regions (Supplementary Figures 1–5, peak coordinates for face-selective regions and scene-selective regions were determined with Neurosynth, https://www.neurosynth.org/). In subsequent analyses, we focused on comparing the performance of different models, first in terms of their overall accuracy (averaged across the entire brain), and then at the level of individual voxels.

### 3.1. Comparing the Average Performance of Different Models

To compare the overall predictive accuracy across different MVPD models, the proportion of variance explained for each model was averaged across the whole brain. Then, we performed pairwise comparisons among all five example models. For each pair of models, we subtracted the variance explained varExpl of one model from that of another one. This procedure yielded a difference value for each participant, and we conducted a one-sample one-tailed *t*-test on the difference values across all 14 participants using SnPM. All *p*-values were Bonferroni corrected for 20 multiple comparisons. Results are shown in Figure 2B as difference matrices for FFA and PPA, respectively.

Overall, models based on artificial neural networks outperformed standard linear regression models. Linear regression based on principal component analysis (**PCA_LR**) showed the worst predictive accuracy while **NN_5layer_dense** proved to be the best predicting model. More precisely, using FFA as seed region, **L2_LR** showed a significantly higher average variance explained than **PCA_LR** [*t*_(13)_ = 6.05, *p* = 0.0004 corrected]. Both **NN_1layer** and **NN_5layer** significantly outperformed **PCA_LR** in terms of average variance explained [NN_1layer: *t*_(13)_ = 6.27, *p* = 0.00028 corrected; NN_5layer: *t*_(13)_ = 6.46, *p* = 0.00022 corrected]. **NN_5layer_dense** revealed a significantly higher average variance explained than **L2_LR** [*t*_(13)_ = 7.72, *p* <0.0002 corrected] and **PCA_LR** [*t*_(13)_ = 6.37, *p* = 0.00024 corrected]. Using PPA as seed region, all the other models showed significantly better predictive performance than **PCA_LR** [L2_LR: *t*_(13)_ = 6.55, *p* <0.0002 corrected; NN_1layer: *t*_(13)_ = 5.79, *p* = 0.00062 corrected; NN_5layer: *t*_(13)_ = 5.49, *p* = 0.00104 corrected; NN_5layer_dense: *t*_(13)_ = 6.68, *p* <0.0002 corrected]. In addition, **NN_5layer_dense** also significantly outperformed **L2_LR**, **NN_1layer** and **NN_5layer** [L2_LR: *t*_(13)_ = 6.30, *p* = 0.00028 corrected; NN_1layer: *t*_(13)_ = 3.45, *p* = 0.04308 corrected; NN_5layer: *t*_(13)_ = 3.73, *p* = 0.02522 corrected]. The rest of the pairwise comparisons did not show significant differences across participants (*p*> 0.05 corrected).

### 3.2. Comparing the Performance of Different Models at the Level of Individual Voxels

**Figure 3:**
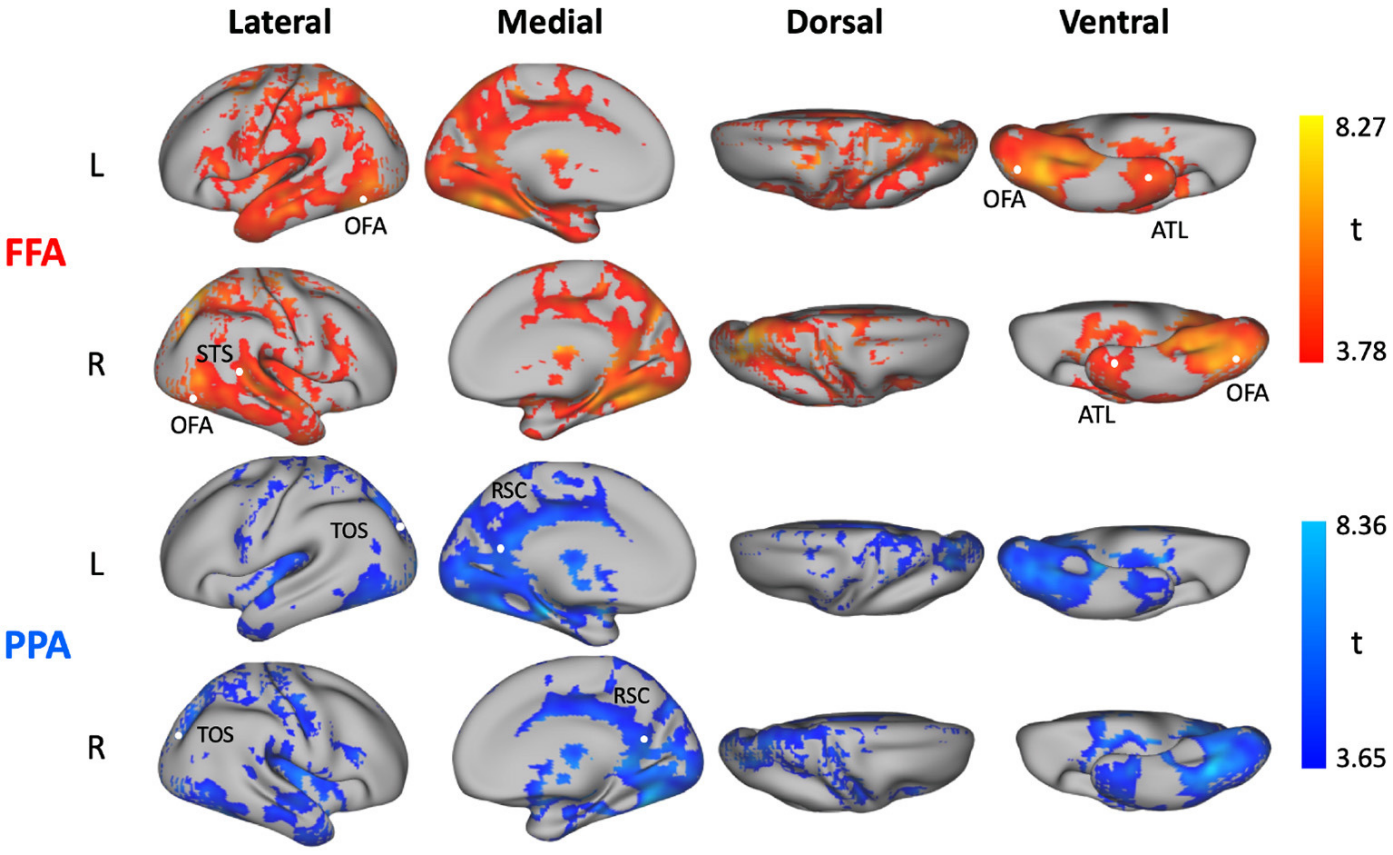
Comparison between neural network (NN) models and linear regression (LR) models. Statistical t-maps computed across subjects from the voxelwise difference between the average variance explained predicted by three NN models (**NN_1layer, NN_5layer, NN_5layer_dense**), and the average variance explained predicted by two LR models (**L2_LR, PCA_LR**) with FFA (top) and PPA (bottom) as predictor ROIs, respectively. The SnPM threshold corrected at *p* <0.05 FWE is 3.78 using FFA as predictor and is 3.65 using PPA as predictor.

To further understand the relative accuracy of different models in different brain regions, we tested the relative performance of neural network models (NN) to the performance of linear regression (LR) models. In particular, we averaged the variance explained for each voxel across the three NN models (**NN_1layer**, **NN_5layer**, **NN_5layer_dense**), and the two LR models (**L2_LR**, **PCA_LR**), respectively. Given the higher predictive accuracy of NN models over LR models when averaged across the whole brain (Figure 2B), we also expected NN models to outperform LR models in several brain regions. We tested this hypothesis by calculating the difference in predictive accuracy between average NN and LR models for each voxel in each participant, and then computed the statistical significance across participants. As expected, the resulting SnPM t-map shown in Figure 3 revealed a large portion of the gray matter that was better predicted by the average NN models rather than the average LR models using FFA or PPA as seed region. NN models did not achieve higher predictive accuracy in the seed regions—this is to be expected, since a very simple model such as the identity function would be sufficient in these regions. By contrast, responses in the other category-selective regions (i.e., face-selective regions: OFA, STS, ATL; scene-selective regions: RSC, TOS) were better predicted by the average NN models over the average LR models when using the seed region of the matching category (Figure 3).

**Figure 4:**
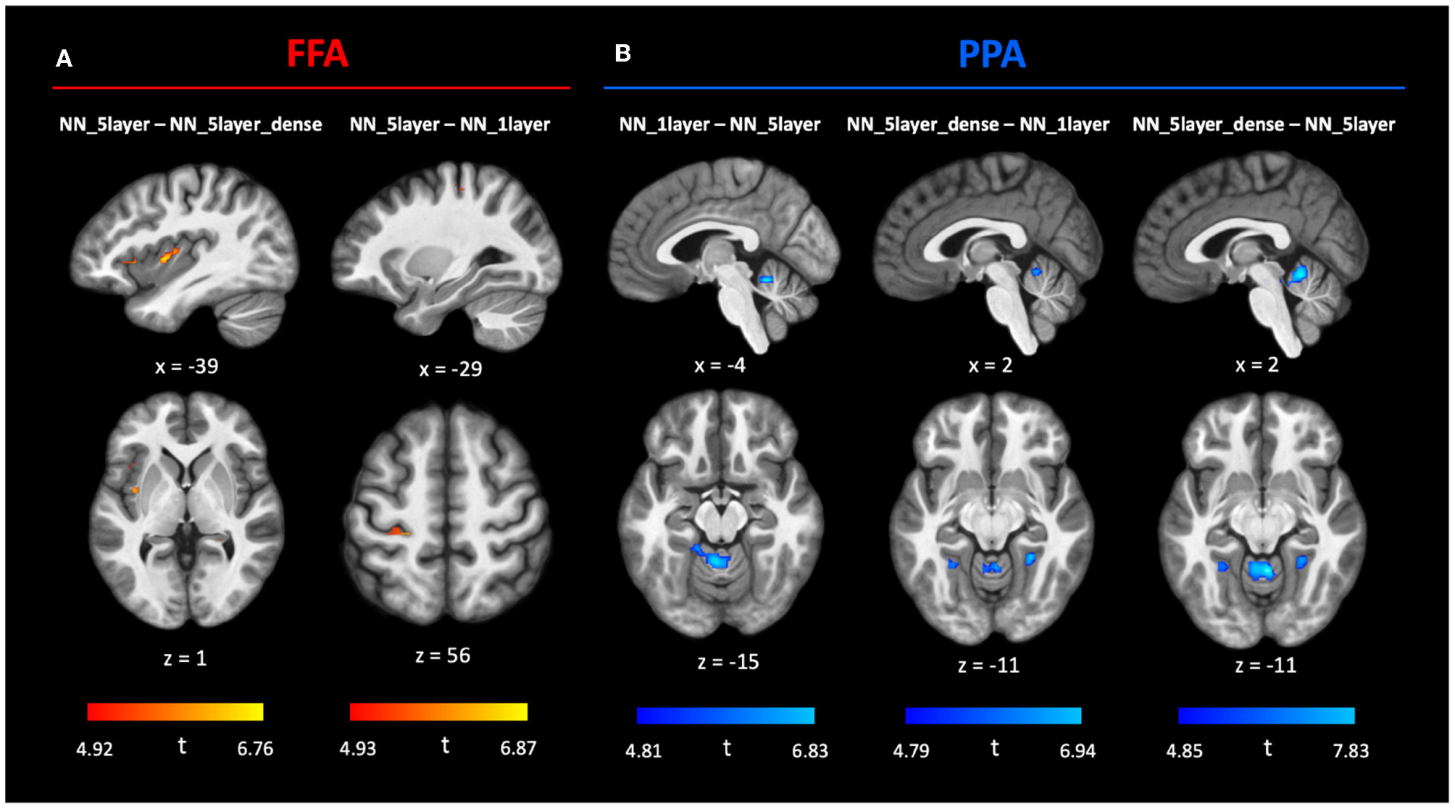
Comparison between MVPD neural network (NN) models. Statistical t-maps computed across subjects from the pairwise difference between the variance explained predicted by three neural network models (**NN_1layer, NN_5layer, NN_5layer_dense**) with FFA **(A)** and PPA **(B)** as predictor ROIs, respectively. The SnPM *p*-values were Bonferroni corrected for all 6 comparisons. We showed interesting brain regions that were better predicted by one NN model than the other NN model at *p* <0.05 FWE after Bonferroni correction. The full NN model comparison results can be found in Supplementary Table 1 (FFA) and Supplementary Table 2 (PPA).

Next, we investigated in more detail the relative voxel-wise predictive accuracy among the three NN models. To do this, we calculated the difference values of variance explained between each pair of NN models (6 pairs in total). Statistical significance was computed using SnPM and all *p*-values were Bonferroni corrected for 6 multiple comparisons. Due to controlling for multiple comparisons both across voxels (with a FWE-corrected voxelwise threshold determined with SnPM) and across multiple model comparisons (thus further dividing the threshold by 6), this analysis is very stringent. Nonetheless, the analysis did reveal some loci of significant differences between the models (Figure 4). Using FFA as seed region, the insula was significantly better predicted by **NN_5layer** over **NN_5layer_dense** (Figure 4A), and a region in left parietal cortex was significantly better predicted by **NN_5layer** over **NN_1layer** (Figure 4B). Using PPA as seed region, a region in the cerebellum showed significant higher predictive accuracy by **NN_1layer** than **NN_5layer**, by **NN_5layer_dense** than **NN_1layer**, and by **NN_5layer_dense** than **NN_5layer**. Additional, smaller loci showing significant differences are reported in Supplementary Table 1 (FFA) and Supplementary Table 2 (PPA).

Finally, since qualitative differences that do not pass significance might still be helpful for users interested in choosing a model, we generated a map that visualizes the best performing model for each voxel, and the extent to which the best model outperforms the other models (Supplementary Figure 6). Specifically, we assigned different colors to each model (**L2_LR**: green; **PCA_LR**: blue; **NN_1layer**: red; **NN_5layer**: yellow; **NN_5layer_dense**: purple). The color of each voxel was set to the color of the model that performed best at predicting that voxel's responses, and the color's saturation was set proportionally to the lowest *t*-value from all pairwise comparisons between models. In other words, more saturated colors appear in voxels for which the difference between the best model and the runner-up model is greater. Together, the voxelwise analyses revealed that there isn't a single best model for all voxels, instead, different voxels are best predicted by different models.

## 4. Discussion

In this article, we have introduced PyMVPD, a Python-based toolbox for multivariate pattern dependence (MVPD). MVPD is a novel technique that investigates the statistical relationship between the responses in different brain regions in terms of their multivariate patterns of response (Anzellotti et al., 2017a). Previous studies have shown that this approach brings higher sensitivity in detecting statistical dependence than standard functional connectivity (Anzellotti et al., 2017a,b). However, given the complex nature of the analysis, the implementation of MVPD can be an obstacle to its wider application. PyMVPD enables researchers to perform complex MVPD analyses with a few lines of easily readable Python code, therefore, it makes MVPD more accessible to a broader community of researchers.

PyMVPD provides users with a flexible analysis framework to study the multivariate statistical dependence between brain regions. Users can choose whether or not to use dimensionality reduction, and if dimensionality reduction is selected, PyMVPD offers a choice between principal component analysis (PCA) and independent component analysis (ICA). Furthermore, PyMVPD permits the use of a variety of models to study the multivariate statistical dependence between brain regions. In addition to the standard linear regression models that have proven to be effective in the previous literature (Anzellotti et al., 2017a), we make artificial neural networks available for connectivity research through an integration with PyTorch. The artificial neural network version of MVPD implemented in PyTorch is introduced for the first time in this article. We demonstrate that the neural network implementations of MVPD outperform the previously published version based on PCA in most brain regions. Example code is provided for three neural network architectures. In addition, users can choose other architectures with different numbers of hidden units and of layers by changing the parameter settings. For applications that require models beyond the family of options already available in PyMVPD, the toolbox is designed so that it is straightforward to program custom architectures and to integrate them with the other scripts.

In the experimental applications described in this work, we tested PyMVPD using the StudyForrest dataset, with the FFA and PPA as seed regions. The results revealed interactions between these seed regions and the rest of the brain during movie-watching, following a pattern that is consistent with the previous literature. Category-selective peaks identified with Neurosynth fell within the MVPD maps for the corresponding category. Overall, artificial neural networks outperformed linear regression models in terms of the predictive accuracy for statistical dependence. Importantly, this is not a trivial consequence of the fact that the artificial neural networks are more complex. In fact, MVPD trains and tests models with independent subsets of the data, and models with more parameters do not necessarily perform better at out-of-sample generalization.

Interestingly, no single model outperformed all others in every voxel. In particular, the **NN_5layer** outperformed other models at predicting responses in the insula and parietal regions using the FFA seed as predictor. By contrast, **NN_5layer_dense** outperformed other models at predicting cerebellar responses given PPA inputs. A qualitative analysis revealed large, contiguous cortical regions in which one model type outperformed the others (Supplementary Figure 6). Taken together, these results indicate that the statistical dependence between different sets of regions might be best characterized by different models. Why would this be the case? It is expected that the interactions between different sets of brain regions implement different kinds of computations. For example, the computations implemented by the interaction between the fusiform face area (FFA) and the occipital face area (OFA)—hypothesized to be upstream of FFA in a hierarchy of visual processing—are likely to be different from the computations implemented by the interaction between the FFA and frontal cortex regions involved in attention. We hypothesize that such differences in the underlying computations could lead to differences in terms of which neural network architectures yield the best models of between-region interactions.

The present results have broader implications for the study of statistical dependence between brain regions: in the literature on brain connectivity, the focus has been largely placed on whether or not two brain regions interact. However, a key direction for future research consists in investigating not only whether two regions interact, but also how they interact. The observation that the statistical dependence between the seed regions and different voxels were best captured by different models suggests that PyMVPD could be used to make progress in this direction.

To pursue goals such as this, PyMVPD is designed to be easily customized and extended. In addition to the five example models (i.e., **L2_LR**, **PCA_LR**, **NN_1layer**, **NN_5layer**, **NN_5layer_dense**) implemented in this article, PyMVPD allows users to build their own MVPD models with customized function components as well as evaluation metrics, making this toolbox an ideal environment to compare the predictive accuracy of different types of models to study the interactions between brain regions.

Installing the full version of PyMVPD requires a working installation of PyTorch, installed compatibly with the version of the CUDA drivers of the GPUs. For users who prefer to avoid this step and do not need to use the neural networks, we make available the LITE version of PyMVPD, that includes only the linear regression models, and does not require PyTorch. The LITE version can be also installed using the Python Package Index (with “pip”).

The toolbox offers a variety of different models that can be used to characterize the interactions between brain regions. The selection of a model among the available options can be based on multiple considerations. First, in this study, we found that artificial neural network models were more accurate than the PCA-based linear regression and the L2 linear regression overall. For this reason, when analyzing a comparable amount of data, and when maximum accuracy is needed, we recommend using artificial neural networks. However, models using artificial neural networks require a working Pytorch installation, and the additional accuracy they offer might not be needed for some use cases. In addition, it is essential to note that there is a trade-off between model complexity and model fit: more complex models may not perform well when the amount of data is limited. For this reason, when a smaller number of volumes is available for training, we recommend using the L2 linear regression (Ridge Regression) model, as it offers the additional flexibility of setting the regularization parameter appropriately for the amount of data available. We also note that the optimal model choice may depend not only on the amount of available data, but also on the amount of noise in the data. For this reason, in cases where maximizing the accuracy is essential, we recommend using data from a small subset of participants to test and compare multiple different model choices. The best performing model can then be used to analyze data from the left-out participants. To avoid circularity in the analyses, it is essential to ensure that the data used to select the optimal model are not later reused to estimate the variance explained by that model.

Together with both versions of PyMVPD, we provide step-by-step tutorials on how to calculate MVPD using the toolbox (https://github.com/sccnlab/PyMVPD/blob/main/exp/PyMVPD_Tutorial.ipynb, https://github.com/sccnlab/PyMVPD_LITE/blob/main/exp/PyMVPD_LITE_Tutorial.ipynb). The tutorials are written with Jupyter Notebook, and include sample data as well as the option to plot one's results side by side with the results we computed. This will make it easier for users to check that the toolbox was installed correctly and to confirm that the results match with those we obtained.

Despite the several options available in PyMVPD, the toolbox still has several limitations. For example, functions to automatically select the optimal number of dimensions from the data when using dimensionality reduction have not yet been implemented. In addition, while PyMVPD offers a variety of neural network architectures, including standard feedforward neural networks and DenseNets, other architectures (such as ResNets) are not available, and would require users to develop their own custom code, which can be integrated with the rest of the toolbox. Importantly, we note that the scope of the toolbox is restricted to multivariate analyses of statistical dependence based on MVPD, and as such it does not include other multivariate measure of statistical dependence, univariate measures of statistical dependence such as functional connectivity, nor other multivariate analyses such as decoding or representational similarity analysis. For such analyses, there are several other existing toolboxes that can be used. In particular, users interested in univariate analyses of connectivity may use the Conn toolbox (Whitfield-Gabrieli and Nieto-Castanon, 2012) or GraphVar (Kruschwitz et al., 2015), and users interested in multivoxel pattern analysis (MVPA), including multivariate decoding and representational similarity analysis, may use the PyMVPA toolbox (Hanke et al., 2009), the CoSMoMVPA toolbox (Oosterhof et al., 2016), or “the decoding toolbox” (tdt, Hebart et al., 2015).

A common criticism of methods based on artificial neural networks is that they operate as a black box: it can be difficult to interpret how the neural networks work in terms of cognitively relevant dimensions. Fortunately, an increasing number of techniques are being developed to improve the interpretability of artificial neural networks (Zhang and Zhu, 2018; Li et al., 2021). While additional work will be needed to integrate these techniques with MVPD, the current MVPD framework based on artificial neural networks already offers the benefit of more sensitive detection of statistical dependence as compared to regularized regression, and the opportunity to compare the performance of different model architectures.

The present study focused on the FFA and PPA as seed regions because they have been studied in depth in previous literature. Future studies can extend our results, investigating the application of PyMVPD to other seed regions. The current implementation of PyMVPD is based on simultaneous prediction: responses in the target region at a given time are predicted from responses in the predictor region at the same time. However, other researchers could take advantage of the customization options to use the responses in multiple timepoints in the predictor region to predict the responses in the target region at each timepoint. Finally, the models of statistical dependence implemented by PyMVPD are deterministic. Multivariate probabilistic models that capture the distribution of uncertainty in predictions are in principle possible, but would require large amounts of data for training.

Although PyMVPD was specifically developed for fMRI analysis, the generic design of the framework makes it widely applicable to other data acquisition modalities (i.e., EEG, MEG) across a variety of domains of brain imaging research. We hope that this toolbox removes some of the barriers to the adoption of MVPD, and facilitates the diffusion of multivariate analyses of the interactions between brain regions.

## Data Availability Statement

The fMRI data used in this study can be obtained from https://www.studyforrest.org. The PyMVPD toolbox code is available at https://github.com/sccnlab/PyMVPD. Further inquiries can be directed to the corresponding author/s.

## Ethics Statement

The studies involving human participants were reviewed and approved by Otto-von-Guericke University. The patients/participants provided their written informed consent to participate in this study.

## Author Contributions

MF, CP, and SA: study conception, design, analysis, and interpretation of results. MF and SA: toolbox development and draft manuscript preparation. All authors reviewed the results and approved the final version of the manuscript.

## Funding

This work was supported by a Startup Grant from Boston College and by NSF Grant 1943862 to SA.

## Conflict of Interest

The authors declare that the research was conducted in the absence of any commercial or financial relationships that could be construed as a potential conflict of interest.

## Publisher's Note

All claims expressed in this article are solely those of the authors and do not necessarily represent those of their affiliated organizations, or those of the publisher, the editors and the reviewers. Any product that may be evaluated in this article, or claim that may be made by its manufacturer, is not guaranteed or endorsed by the publisher.
