# Supplementary material for: PyMVPD: A Toolbox for Multivariate Pattern Dependence
Source: Front Neuroinform. 2022 Jun 23;16:835772. doi: 10.3389/fninf.2022.835772 (PMC9262406; doi:10.3389/fninf.2022.835772)
Supplement: Supplementary file 1 [file Data_Sheet_1.pdf]

Supplementary Materials

Supplementary Figures

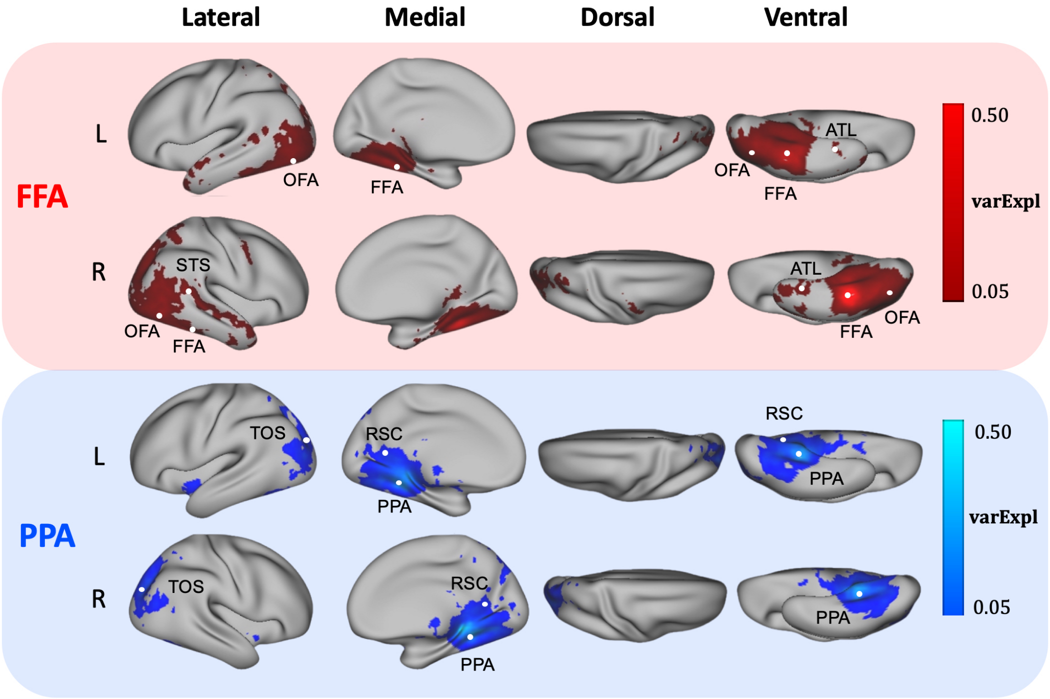

Figure S1: MVPD profiles of the seed regions computed by the **L2_LR** model. The proportion of variance explained varExpl obtained by PyMVPD using data from FFA (red) and PPA (blue) as predictors was averaged across all 14 participants and was thresholded at 5%.

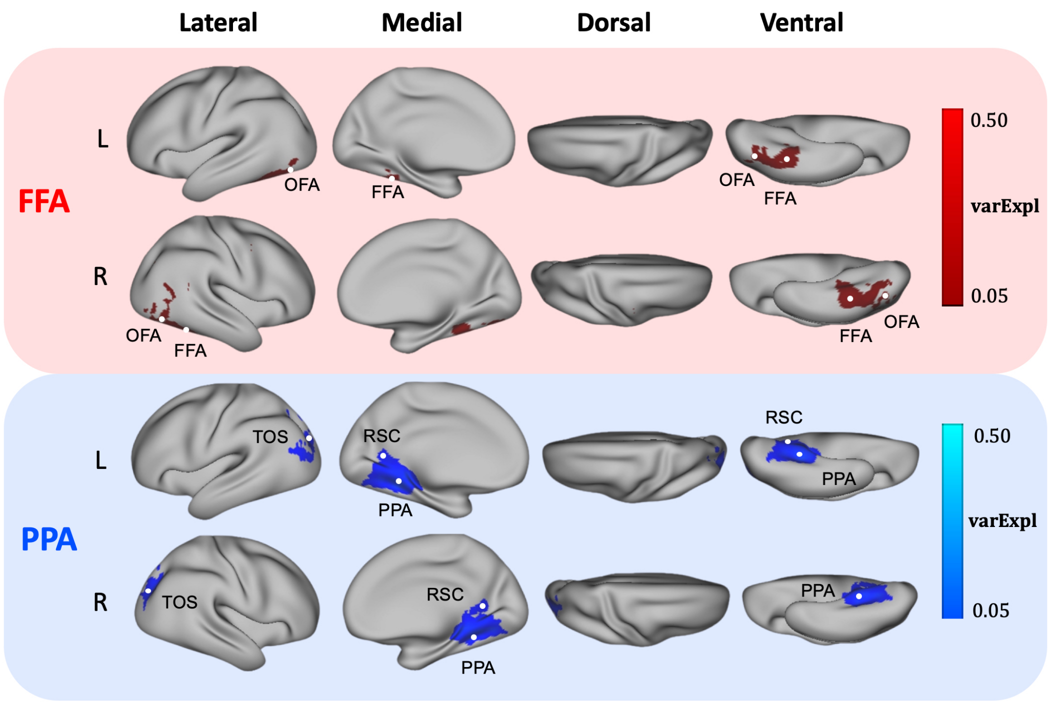

Figure S2: MVPD profiles of the seed regions computed by the **PCA_LR** model. The proportion of variance explained varExpl obtained by PyMVPD using data from FFA (red) and PPA (blue) as predictors was averaged across all 14 participants and was thresholded at 5%.

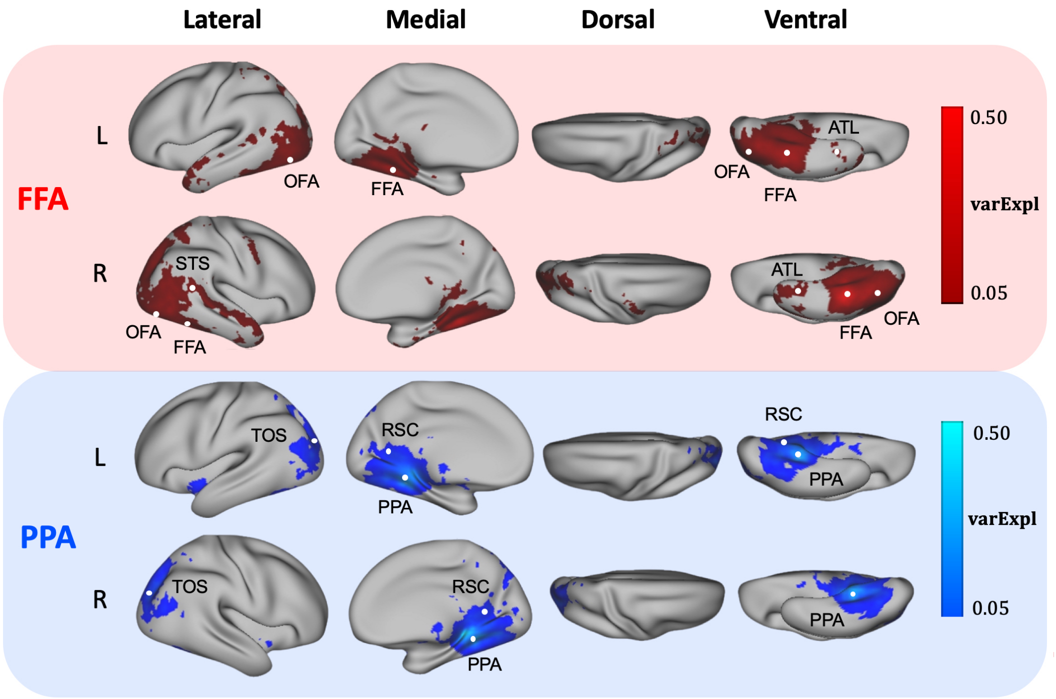

Figure S3: MVPD profiles of the seed regions computed by the **NN_1layer** model. The proportion of variance explained varExpl obtained by PyMVPD using data from FFA (red) and PPA (blue) as predictors was averaged across all 14 participants and was thresholded at 5%.

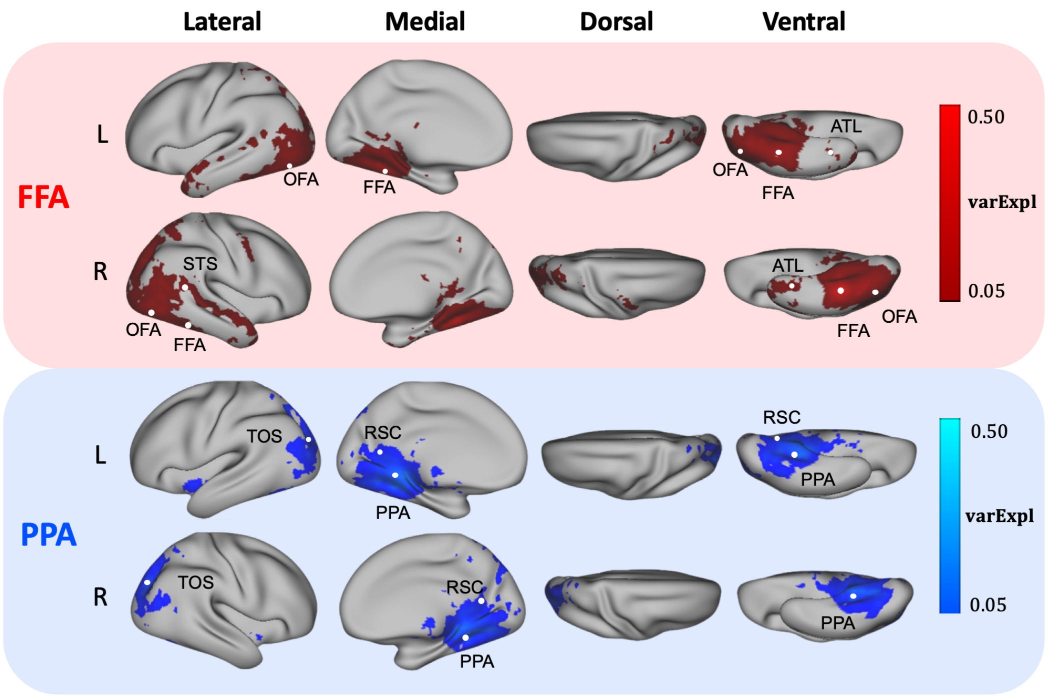

Figure S4: MVPD profiles of the seed regions computed by the **NN_5layer** model. The proportion of variance explained varExpl obtained by PyMVPD using data from FFA (red) and PPA (blue) as predictors was averaged across all 14 participants and was thresholded at 5%.

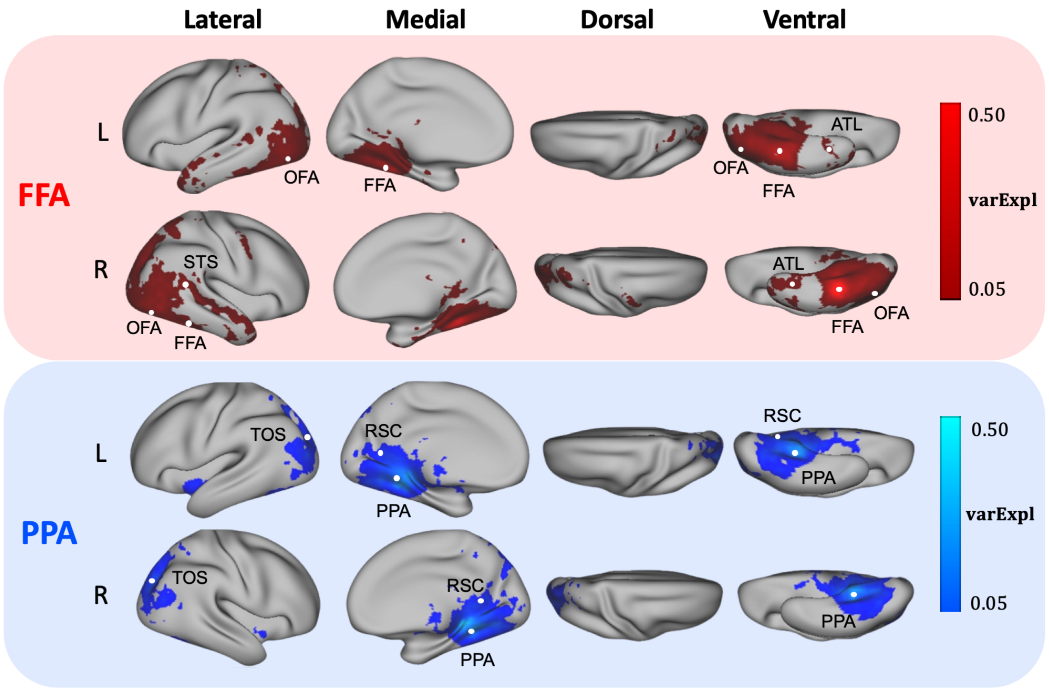

Figure S5: MVPD profiles of the seed regions computed by the **NN_5layer_dense** model. The proportion of variance explained varExpl obtained by PyMVPD using data from FFA (red) and PPA (blue) as predictors was averaged across all 14 participants and was thresholded at 5%.

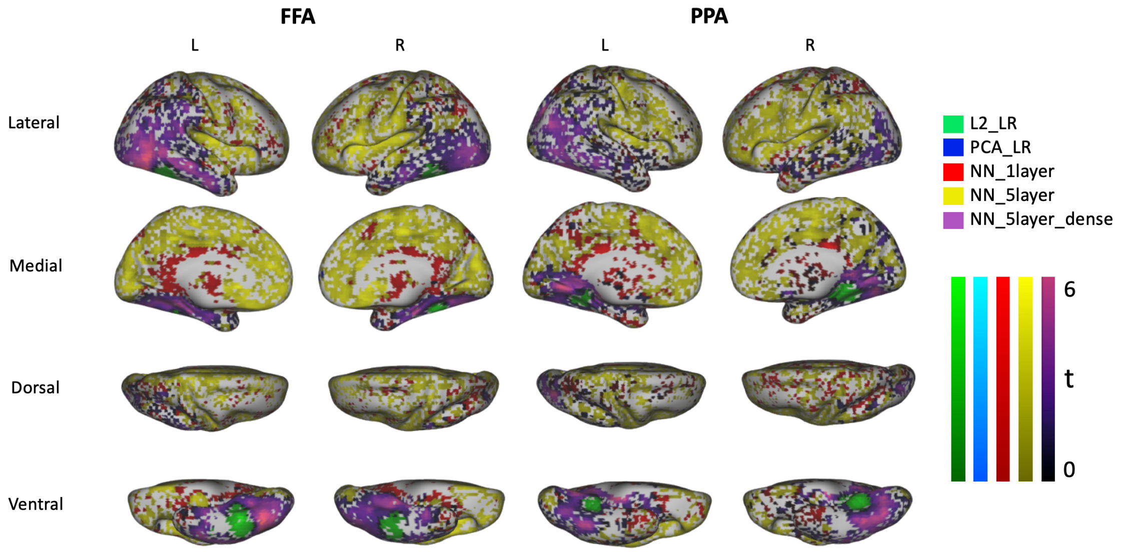

Figure S6: **Voxelwise optimal models.** For each voxel, the brain maps are colored according to the MVPD model that provides the highest predictive accuracy using seed regions as FFA (left panel) or PPA (right panel) in that voxel. Color saturation was set as the lowest t-value from all pairwise comparisons between the best-predicting model for that voxel and other models, reflecting the difference between the best model and the second best model. Distinct, contiguous portions of cortex are best predicted by different model types (green: L2_LR, blue: PCA_LR, red: NN_1layer, yellow: NN_5layer, purple: NN_5layer_dense).

	NN model comparison	cluster size	voxel-level			MNI coordinates (x, y, z mm)
		k	$P_{FWE-corr+Bonf}$	$P_{FWE-corr}$	$P_{seudo-t}$	
FFA	1layer – 5layer	18	0.0012	0.0002	7.00	(-21,-30,-25)
		20	0.0240	0.0040	5.83	(42,-48,-25)
	1layer – 5layer_dense	2	0.0108	0.0018	6.31	(-9,-36,77)
		28	0.0120	0.0020	6.23	(-12,-72,8)
		12	0.0120	0.0020	6.18	(-3,57,34)
		20	0.0132	0.0022	6.16	(-6,-3,11)
		11	0.0168	0.0028	6.04	(45,-3,-9)
		32	0.0240	0.0040	5.90	(3,-75,14)
		19	0.0276	0.0046	5.88	(15,-33,11)
		12	0.0336	0.0056	5.79	(3,-21,8)
	5layer – 1layer	22	0.0012	0.0002	6.87	(-21,-33,57)
		5	0.0060	0.0010	6.29	(48,-6,11)
		7	0.0072	0.0012	6.21	(-57,-6,18)
		11	0.0336	0.0056	5.77	(30,-6,-9)
		3	0.0372	0.0062	5.71	(-12,-96,-2)
		5	0.0456	0.0076	5.65	(-9,54,37)
	5layer – 5layer_dense	165	0.0048	0.0008	6.76	(-39,-3,4)
		44	0.0048	0.0008	6.74	(-6,3,1)
		42	0.0156	0.0026	6.29	(-3,57,34)
		75	0.0156	0.0026	6.28	(0,-72,14)
		61	0.0228	0.0038	6.02	(24,-36,4)
		17	0.0252	0.0042	5.97	(45,-3,-9)
		72	0.0264	0.0044	5.94	(0,42,11)
		7	0.0288	0.0048	5.91	(3,21,-12)
	5layer_dense – 1layer	39	0.0024	0.0004	6.94	(42,-48,-22)
	5layer_dense – 5layer	39	0.0048	0.0008	6.91	(42,-48,-22)

Table S1: **Comparison between MVPD neural network (NN) models with FFA as the predictor.** SnPM statistical results computed across subjects from the pairwise difference between the variance explained predicted by three neural network models (**NN_1layer, NN_5layer, NN_5layer_dense**) with FFA as the predictor ROI. The SnPM p-values were Bonferroni corrected for all 6 comparisons. The peak of each brain cluster that were better predicted by one NN model than the other NN model at $p < 0.05$ FWE after Bonferroni correction were listed in the table.

	NN model comparison	cluster size	voxel-level			MNI coordinates (x, y, z mm)
		k	$P_{FWE-corr+Bonf}$	$P_{FWE-corr}$	$P_{seudo-t}$	
**PPA**	1layer – 5layer	196	0.0012	0.0002	6.83	(-3,-51,-15)
		35	0.0024	0.0004	6.73	(33,12,-25)
		33	0.0060	0.0010	6.09	(27,-42,-12)
		10	0.0060	0.0010	5.99	(-33,9,-19)
	1layer – 5layer_dense	18	0.0444	0.0074	5.52	(3,-18,34)
	5layer – 1layer	–	–	–	–	–
	5layer – 5layer_dense	12	0.0300	0.0050	5.85	(33,-24,14)
	5layer_dense – 1layer	46	0.0036	0.0006	6.94	(27,-42,-12)
		66	0.0048	0.0008	6.50	(3,-51,-9)
		36	0.0084	0.0014	6.12	(-27,-48,-12)
	5layer_dense – 5layer	171	0.0012	0.0002	7.53	(3,-51,-9)
		45	0.0012	0.0002	7.08	(27,-42,-12)
		40	0.0060	0.0010	6.37	(-27,-48,-12)
		10	0.0276	0.0046	5.88	(-18,-21,-22)
		3	0.0408	0.0068	5.66	(15,3,-22)

Table S2: **Comparison between MVPD neural network (NN) models with PPA as the predictor.** SnPM statistical results computed across subjects from the pairwise difference between the variance explained predicted by three neural network models (**NN_1layer, NN_5layer, NN_5layer_dense**) with PPA as the predictor ROI. The SnPM p-values were Bonferroni corrected for all 6 comparisons. The peak of each brain cluster that were better predicted by one NN model than the other NN model at $p < 0.05$ FWE after Bonferroni correction were listed in the table.
